# Estimating antimalarial drugs consumption in Africa before the switch to artemisinin-based combination therapies (ACTs)

**DOI:** 10.1186/1475-2875-6-91

**Published:** 2007-07-10

**Authors:** Jean-Marie Kindermans, Daniel Vandenbergh, Ed Vreeke, Piero Olliaro, Jean-Pierre D'Altilia

**Affiliations:** 1 AEDES Foundation, 34, rue Joseph II, 1000, Brussels, Belgium; 2 Médecins Sans Frontières, 94, rue Dupré, 1090, Brussels, Belgium; 3 UNICEF/UNDP/WB/WHO Special Programme for Research & Training in Tropical Diseases (TDR), 2, avenue Appia, CH1211 Geneva 27, Switzerland

## Abstract

**Background:**

Having reliable forecasts is critical now for producers, malaria-endemic countries and agencies in order to adapt production and procurement of the artemisinin-based combination treatments (ACTs), the new first-line treatments of malaria. There is no ideal method to quantify drug requirements for malaria. Morbidity data give uncertain estimations. This study uses drug consumption to provide elements to help estimate quantities and financial requirements of ACTs.

**Methods:**

The consumption of chloroquine, sulphadoxine/pyrimethamine and quinine both through the private and public sector was assessed in five sub-Saharan Africa countries with different epidemiological patterns (Senegal, Rwanda, Tanzania, Malawi, Zimbabwe). From these data the number of adult treatments per capita was calculated and the volumes and financial implications derived for the whole of Africa.

**Results:**

Identifying and obtaining data from the private sector was difficult. The quality of information on drug supply and distribution in countries must be improved. The number of adult treatments per capita and per year in the five countries ranged from 0.18 to 0.50. Current adult treatment prices for ACTs range US$ 1–1.8. Taking the upper range for both volumes and costs, the highest number of adult treatments consumed for Africa was estimated at 314.5 million, corresponding to an overall maximum annual need for financing ACT procurement of US$ 566.1 million. In reality, both the number of cases treated and the cost of treatment are likely to be lower (projections for the lowest consumption estimate with the least expensive ACT would require US $ 113 million per annum).

There were substantial variations in the market share between public and private sources among these countries (the public sector share ranging from 98% in Rwanda to 33% in Tanzania).

**Conclusion:**

Additional studies are required to build a more robust methodology, and to assess current consumptions more accurately in order to better quantify volumes and finances for production and procurement of ACTs.

## Background

Reliable forecasts for antimalarial treatments requirements become particularly urgent now that malaria endemic countries are changing their treatment policies and adopting artemisinin-based combination therapies (ACTs) following WHO recommendations [1]. Coverage so far has been low for a variety of reasons, including the lack of reliable forecasts of antimalarial drug volumes needed for both global and local consumption.

More accurate forecasts are, therefore, needed both at the producer's and the customer's end (at country and international level) in order to harmonize supply and demand.

Both morbidity and consumption methods of quantification have limitations, but can be complementary. Morbidity-based estimations of drug requirements do not take into account the actual consumption, which results from drug availability and access, customer's willingness to pay (which largely depends on the treatment price to the end user) and current practice to treat fever (as opposed to parasitologically confirmed malaria) with antimalarial drugs (some 2.84 billion fever episodes would occur annually in Africa [2]). While this latter practice leads to significant over-treatment and wastage, weaknesses in the systems result in the failure to reach patients who do have malaria and need treatment.

Health information systems are insufficient to quantify the requirements for antimalarials [3]. Estimates of the malaria burden vary greatly with the methodology used and assumptions made from 215 to 374 million [4] in Africa (~70% of all cases of falciparum malaria worldwide). To complement malaria morbidity-based forecasts this study used a consumption approach to produce forecasts of volumes (primarily for drug manufacturers and procurement agencies) and financial requirements (of primary interest for donors and governments). In the absence of a standard methodology, five African countries representing diverse geographical and epidemiological settings were selected, and then projections were made based on the data obtained to the whole of Africa, estimating lower and upper levels of consumption.

## Methods

The analysis was limited to first-line drugs in use based on the treatment guidelines at the time of the study, essentially chloroquine and sulphadoxine/pyrimethamine for uncomplicated malaria, and quinine for more severe malaria. A treatment policy change occurred only in Rwanda during the observation period and was taken into account [5].

Representative countries of the main geographical and climatic regions (Southern, Eastern, Central, and Western Africa) were selected, with different endemicities, health systems (relative importance of public and private sector), and treatment-seeking behaviours.

Data were collected in 2003 in Rwanda (Central Africa), Senegal (Western Africa), Tanzania (Eastern Africa), Malawi and Zimbabwe (Southern Africa). The study covered the period 1999–2002. Data were available for the whole period for Malawi and Zimbabwe, while for Rwanda complete information, including the private sector, was available for 2001 and 2002. Data from Senegal were available only for the year 2000 for the public sector, and 2002 for the private sector. For Tanzania, calculations were based on 2000 and 2001 data.

Data were collected through visits, interviews of people in charge of supply or distribution, inspection of internal documents (e.g. distribution list of public central medical stores, list of imports of private wholesalers), source verification, assisted by national and international consultants for this specific study. Both the public and private sectors were investigated. The informal sector was not analysed systematically.

More specifically, imports, sales or deliveries of the agencies importing antimalarial medicines were evaluated.

In the public sector, investigations concerned the central medical store (or equivalent), the public services which procure or receive antimalarial drugs directly (e.g. National Malaria Control Programme), and international organizations which might be involved in the procurement or donation of antimalarial drugs. The semi-public sector (private non-profit and faith-based organizations) was considered also as part of the public sector. For the private commercial sector, all the local wholesalers were contacted (or, if too many, the main ones, targeting 80% of the turnover).

Production from domestic manufacturers was also taken into account if aimed for the domestic market, unless already accounted for in the estimates from public and private sectors.

All these agencies and companies were identified in collaboration with the national public health and pharmaceutical authorities, and a list of the organizations to be visited was further prepared. The market share of the surveyed organizations in the private and the public sector was estimated through the analysis at the national level of import data, pharmacy council data and interviews of local officials and experts. When the turnover estimate was below 100% because of non-responders, a post-hoc extrapolation was made in order to estimate the total demand in each sector.

For each identified drug, the consumption was studied for all dosages and forms on the market: adult and paediatric dosages, oral (solid and syrups) and injectable forms. International Non-proprietary Names (INN) were used instead of brand names.

Data were collected first about the smallest pharmaceutical unit. They were then aggregated by pharmaceutical form for each drug, subsequently by drug, then by sector, and finally by country.

All quantities, including paediatric tablets, were transformed into equivalent adult treatments. The conversion formulas used were based on the WHO recommended adult malaria treatment course [6]. Syrup formulations and injectable formulations were not converted to equivalent adult treatment courses, but this has a negligible impact on overall volumes. It was not possible to separate use for treatment and prophylaxis, but the latter represents a marginal part of the consumption in studied countries according to information collected within national malaria programmes (especially at the time when data were collected). For injectable drugs, the indicator was the quantity of ampoules required for a one-day adult treatment.

Consumption was systematically annualized in each country. Indeed, in the public sector, distribution to peripheral stores was most often recorded monthly, while supply was done much less frequently (e.g. through tenders). In the private sector imports are more frequent than in the public sector, which allowed calculating monthly mean evaluations of imports, before annualizing.

The number of oral treatments of malaria consumed in Africa were projected based on the general population data and forecasts for the continent [7], together with the data on percentage of population at malaria risk [8]. The population at risk was calculated in Africa for 2004. Based on available figures, people at risk of endemic and epidemic malaria were calculated at 566,283,000 in 2000, and 629,030,000 in 2004. The estimated population at risk for the whole of Africa was then multiplied by the upper and lower figures of the consumption of antimalarial medicines (expressed by the ratio of adult treatment per capita), in order to estimate the range of global consumption.

## Results

### Sources of information identified

Identifying and obtaining data from the formal private sector was difficult. A centralized system tracing products provided through both the public and the private sector did not exist in the study countries. National health authorities had scanty information on importers and distributors for the private sector. Equally incomplete were data on imported medicines, which, when available, were aggregates of different compounds, presentations and trade names, lumped together to express value of goods.

Table 1 shows the number of organizations visited in the private and the public sector in each country, as well as their estimated market share.

**Table 1:** Informants visited.

***Country***	***Malawi***		***Rwanda***		***Senegal***		***Tanzania***		***Zimbabwe***	
**Informants**	private	public	private	public	private	public	private	public	private	public

**Importers contacted**	7	2	1	2	3	2	14	1	8	1
**Importers providing data**	6	2	1	2	3	2	8	1	8	1
**Importers declining**	1	0	0	0	0	0	3	0	0	0
**Estimated % of market**	80%	100%	90%	100%	>80%	100%	<50%	100%	80%	100%
**Manufacturers visited**	2		0		2		1		1	
**Estimated % of market**	100%		n.a.		100%		100%		100%	

Private importers and distributors were disinclined to share information about sales, but less reluctant to disclose import data. In contrast, the public sector, including not-for-profit organizations, was considerably more receptive and > 80% of the turnover of this sector was captured.

In general, one can be reasonably confident in the data gathered in Malawi and Zimbabwe (data collected for 1999–2002) as well as Rwanda (complete information, including private sector, for 2001 and 2002). Data from Senegal were more fragmentary, but these figures were in good agreement with another study by Management Science for Health for 2002 [9]. Reliable data were available for the year 2000 for public services, while for 2002, data were obtained only for two companies in the private sector (and incomplete data were to be extrapolated for one of them). In Tanzania calculations were based on the years 2000 and 2001, which were more reliable for public services. Confidence in the figures there is lower than for the other countries due to the large number of private importers, their rather low level of response, and the lack of correlation between their figures and those from the pharmacy board.

Besides the collection of data itself, potential biases in the methodology might originate from other external factors affecting consumption: changes in both the economic and epidemiological patterns influence private and public demand, which was partly minimized by collecting data over several years. Changes in prices of drugs or user-fee system can also affect the results. However, none of them appeared to be significant during the period.

### General findings on consumptions

Table 2 shows the number of solid form oral adult treatments consumed per year and provided by both the public and private sectors during the observation period in each country with respect to the population of the country in 2001 [10].

**Table 2:** Solid form oral treatments yearly consumed in each country (public and private sector).

***Country***	***Malawi***	***Rwanda***	***Senegal***	***Tanzania***	***Zimbabwe***
**Population (millions) [Ref 10]**	11,8	8,4	10,6	35,5	12,7
**Calculated number of adult treatments equivalents (millions)**	4,6	1,5	5,3	15	5,4
**Calculated adult treatments equivalents per capita**	0,39	0,18	0,50	0,42	0,43
**Estimated number of malaria cases (million) [Ref 11]**	5,5	1,2	4,3	14,5	4

In order to compare countries and to extrapolate results to other countries in the continent, the ratio of adult treatment equivalents to the overall population was calculated at between 0.18 and 0.50. The resulting figures in terms of total and per-capita number of adult treatments equivalents are somehow consistent with the WHO estimated number of cases in those countries [11].

### Market share of antimalarial medicines in the private and public sector

**Figure 1:**
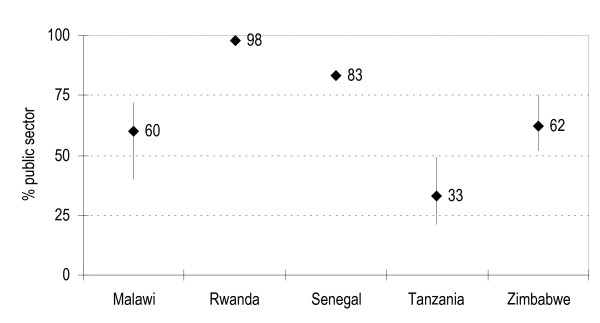
Country percentage share by public sector of antimalarial treatments over the period under study.

Figure 1 shows the proportion of antimalarial oral treatments (except syrups) distributed and sold through the public and the private sector in each country. Tablets represent the vast majority of the antimalarials distributed or sold. Drug supplies by public institutions are dominant in Rwanda (>90%), Senegal (>80%), high in Malawi (ca. 60% on average over four years), and much lower in Zimbabwe (38%) and Tanzania (33%).

By contrast, syrups, which are more expensive, and represent a very small proportion of the total treatments are essentially sold through the private sector (Table 3). For both syrup and injectable formulations there was significant inter-country variation (highest consumption in Senegal and Tanzania). In Senegal the public sector had the largest share of the supplies of injectable treatments (78%), which was related to the prevailing practice of treating uncomplicated malaria with injectable quinine in that country.

**Table 3:** Yearly volumes of syrups and injectables, with public and private sectors market shares.

***country***	***Malawi***	***Rwanda***	***Senegal***	***Tanzania***	***Zimbabwe***
**Number of syrups (units)**	2,750	5,500	295,000	561,000	53,500
**Syrups/total adult treatments**	<0.1%	0.4%	5.5%	3.7%	1%
**% sales by private sector**	50%	50%	84%	100%	80%
**Injectables (1 day treatment equivalents)**	26,000	30,000	954,000	1,000,000	16,000
**% sales by private sector**	60%	50%	22%	75%	100%

### Estimation of requirements for antimalarial medicines in Africa

As stated in the methods section, circa. 629 million were estimated to be at risk of malaria in Africa in 2004. When applying the lower (0.18) and upper (0.50) rates of consumption to this overall population (Table 2), the global consumption of antimalarials in Africa can be estimated in the range of 113 to 314.5 million adult treatments.

Current prices of an ACT adult treatment adopted in African countries vary between US$ 1 (artesunate+amodiaquine, Sanofi-Aventis), and US$ 1.8 (artemether+lumefantrine, Novartis) for public or not-for-profit organizations.

Together, these data lead to an overall yearly financial requirements between US $ 113 million (lower consumption estimate with cheaper ACT) and 566.1 million (higher consumption estimate with most expensive ACT) if the same levels of consumption for chloroquine and sulphadoxine/pyrimethamine apply to ACTs.

## Discussion

The aim of this study was to generate simple, locally collected information to help estimate antimalarial drugs consumption (as orders of magnitude as opposed to accurate estimates) in the recent past in Africa. Malaria treatment policies have changed from monotherapies (used when this study was conducted) to artemisinin-based combination therapies (ACTs) as now recommended by the World Health Organization (WHO) [1,12,13]. So far, lack of reliable data to quantify demand, compounded with the challenges of changing policy and practice, contribute to the current failure to deploy ACTs at the levels required. As a consequence supplies are not adapted and adequate funding for procurement cannot be planned.

This study was done in order to help inform production and procurement of ACTs of potential future demand as well as projecting the corresponding financial requirements based on cost of drugs, while acknowledging that other costs are also involved in policy change. The study has methodological limitations.

In the first place, consumption methods are better suited when current policies and patterns continue unchanged, which is not the present case : ACTs, possibly after parasitological confirmation, are to replace monotherapies taken on clinical ground. Here, in order to complement equally questionable morbidity-based data, consumption generates information based on the number of fever episodes actually treated with antimalarials, whether true malaria cases or not. The consequences on the introduction of ACTs on consumption are difficult to predict. A wider use of rapid diagnostic tests will likely affect consumption. ACTs are more effective but also more expensive than traditional monotherapies. In the short term their cost and limited supplies will hold back scaling-up. Their efficacy may, in the medium term, generate increased demand but in the longer term reduce morbidity, though the effect will largely depend on intensity of transmission.

Another issue is that the choice of study countries was based on epidemiological and not statistical criteria. In addition, the calculations made assumed the price to the end user to be the same of former first-line single-agent treatments, while this may be very different depending on whether ACT will be subsidized or not and to what extent. A case has been made for ACTs to be subsidized [14]. Furthermore, tracking data is inherently difficult within the current system in both the private and public sector, and this is obviously reflected in this study. For instance, detailed information on sales could not be obtained by private importers and distributors essentially because they feared this could be used against them either by the fiscal administration or competitors.

Finally, the informal sector was not specifically investigated. However, using official drug imports into the country should account for it as well, as these retailers generally obtain their medicines from private wholesalers, private pharmacies, or even public services. One must acknowledge though that variable amounts of drugs may enter countries illegally, and this obviously cannot be accounted for.

The fact that extrapolations generated with a consumption approach match and support morbidity-based estimates [4] may be a mere coincidence as the two methods measure different variables: the number of cases that should ideally be treated on the one hand, and the number of treatments used on the other hand.

The other component of this research was financial requirements. While other costs are obviously involved for countries when shifting to ACTs, we have chosen to concentrate on the costs of drugs as the major financial burden. ACTs are more expensive than the traditional antimalarial drugs, and the question is how this will affect penetration and coverage.

This approach estimates financial requirements to range between US $ 113 million (lower consumption estimate with an ACT at US $ 1 per adult treatment) and 566.1 million (higher consumption estimate with an ACT at US $ 1.8 per adult treatment) if the same levels of consumption for chloroquine and sulphadoxine/pyrimethamine apply to ACTs. While this range is very wide, it provides financial brackets which are for instance within reach of international donor support. In comparison, it was estimated that US$ 70 billion were disbursed in official development programmes in 2003 by OECD member states [15].

The next question is by whom these costs will be incurred or covered. This study found a substantial (yet variable) market share of the public sector, which was at odds with the 20% commonly cited for Africa [16-18]. There are several unresolved methodological issues with the collection and interpretation of data on treatment-seeking behaviour which make inference on the drug supplier (private or public) difficult: self-treatment, home treatment, community treatment, treatment by a health professional, are not clearly defined categories and multiple sourcing of treatments is possible. While differences in health-seeking behaviours [19] may contribute to these variations, a plausible explanation is that market shares are usually expressed in financial terms (value of medicines), which tends to overestimate the proportion of drugs supplied through the private market that generally would charge higher unit costs, while here volumes were used instead.

## Conclusion

There were situations in which it was difficult to retrieve and extract information. Countries should improve the quality of information on drug supply and distribution. Better monitoring of the national demand and market will benefit policy makers in order to adapt choices, improve follow up and increase access. Diversity of usage across countries should be considered to adapt projections and interventions.

The results of this study provide information on brackets of volumes and finances for ACTs. Since deployment will be staggered, demand and investments will be more likely to start at the lower end of the spectrum.

The approach used to generate these data should be further tested for its applicability, limitations and robustness in order to further refine it and to assess current consumptions more accurately across Africa.

## Authors' contributions

JMK and JPDA conceived the study, and developed the study design with PO. DV and EV organized and participated in data collection in the different countries, either by visiting countries to collect data or supervising consultants. DV and EV analysed data, with the support of JMK. EV wrote a first draft of results and comments, with support of JMK and JPDA. JMK and PO wrote the manuscript.

The final version of the manuscript was seen and approved by all authors.
